# Pathogenic determinants of *Kingella kingae* disease

**DOI:** 10.3389/fped.2022.1018054

**Published:** 2022-10-11

**Authors:** Eric A. Porsch, Kevin A. Hernandez, Daniel P. Morreale, Nina R. Montoya, Taylor A. Yount, Joseph W. St. Geme

**Affiliations:** ^1^ Department of Pediatrics, Children’s Hospital of Philadelphia, Philadelphia, PA, United States; ^2^ Perelman School of Medicine, University of Pennsylvania, Philadelphia, PA, United States

**Keywords:** *kingella kingae*, type IV pili, adhesin, toxin, capsule, exopolysaccharide, lipopolysaccharide, genomics

## Abstract

*Kingella kingae* is an emerging pediatric pathogen and is increasingly recognized as a leading etiology of septic arthritis, osteomyelitis, and bacteremia and an occasional cause of endocarditis in young children. The pathogenesis of *K. kingae* disease begins with colonization of the upper respiratory tract followed by breach of the respiratory epithelial barrier and hematogenous spread to distant sites of infection, primarily the joints, bones, and endocardium. As recognition of *K. kingae* as a pathogen has increased, interest in defining the molecular determinants of *K. kingae* pathogenicity has grown. This effort has identified numerous bacterial surface factors that likely play key roles in the pathogenic process of *K. kingae* disease, including type IV pili and the Knh trimeric autotransporter (adherence to the host), a potent RTX-family toxin (epithelial barrier breach), and multiple surface polysaccharides (complement and neutrophil resistance). Herein, we review the current state of knowledge of each of these factors, providing insights into potential approaches to the prevention and/or treatment of *K. kingae* disease.

## Introduction

Advances in both culture-based and molecular-based diagnostics have led to increased recognition of *Kingella kingae* as an important pediatric pathogen and have stimulated interest by the research community in understanding the molecular determinants of *K. kingae* pathogenicity. Epidemiological surveillance studies have established that the predominant niche of *K. kingae* is the oropharynx of children ages 6 months to 4 years (1). The organism can be detected in approximately 10% of healthy children in this age range (1–3). In a subset of colonized children, *K. kingae* is able to produce invasive disease, primarily septic arthritis, osteomyelitis, tenosynovitis, and bacteremia and more rarely endocarditis and meningitis (2–4).

For *K. kingae* to produce invasive disease, it must accomplish several critical steps in the pathogenic process. The organism must first adhere to the respiratory epithelium to colonize the oropharynx. After successful colonization, the bacterium must breach the respiratory epithelial barrier and enter the bloodstream. As dissemination to the sites of invasive disease occurs through the hematogenous route, the organism must survive in the hostile intravascular environment and seed a typically sterile body site (e.g., bones, joints, or endocardium) to produce disease.

Over the past two decades, there has been a significant research effort to identify and characterize key factors produced by *K. kingae* that promote steps in the pathogenic process. In this review, we discuss the current state of knowledge of *K. kingae* virulence factors and their potential roles in both colonization and development of invasive disease. By gaining a more thorough understanding of the *K. kingae* factors involved in colonizing the host, transitioning from commensal to pathogen, and invading typically sterile body sites, there is potential to identify novel strategies to prevent or treat invasive *K. kingae* disease.

### Type IV pili

Initial morphological analysis of *K. kingae* with transmission electron microscopy revealed the presence of long, filamentous surface fibers (5). These fibers have been identified as type IV pili (T4P) and are essential for a variety of *K. kingae* virulence-associated phenotypes, including a form of surface motility called twitching motility, natural competence, and adherence to human cells, including respiratory epithelial and synovial cells (6, 7). Early observations of *K. kingae* colony morphology identified two distinct colony types that correlate with T4P expression (5). The spreading/corroding colony type is associated with high levels of surface piliation, and the nonspreading/noncorroding colony type correlates with low levels of surface piliation (5, 6). Subsequent work established that strains lacking pili display a dome-shaped colony type (8). Among clinical isolates*,* the level of piliation varies and can be permanently changed following repeated sub-culture (8, 9). While the majority of oropharyngeal and non-endocarditis blood isolates are piliated, only a small proportion of joint fluid, bone, and endocarditis isolates produce surface pili (8).

*K. kingae* T4P biogenesis depends on production of the major pilin subunit PilA1, which is a ∼15-kDa protein that is exported to the inner membrane and polymerizes to form the T4P filament (10). The PilA1 amino acid sequence varies greatly among clinical isolates, suggesting that this protein experiences selective pressure in the human host (8). Expression of the *pilA1* gene is regulated by the PilS/PilR two-component system and the alternative *σ* factor *σ*54 (10). In prototype strain 269–492, deletion of the *pilR* gene encoding the PilR response regulator and the *rpoN* gene encoding *σ*54 results in abrogation of PilA1 production and the absence of surface pili (10). In contrast, deletion of the *pilS* gene encoding the PilS sensor kinase results in only a partial decrease in PilA1 production and low levels of surface pili, suggesting that the PilR response regulator may remain active in the absence of the PilS sensor (10).

**Figure 1:**
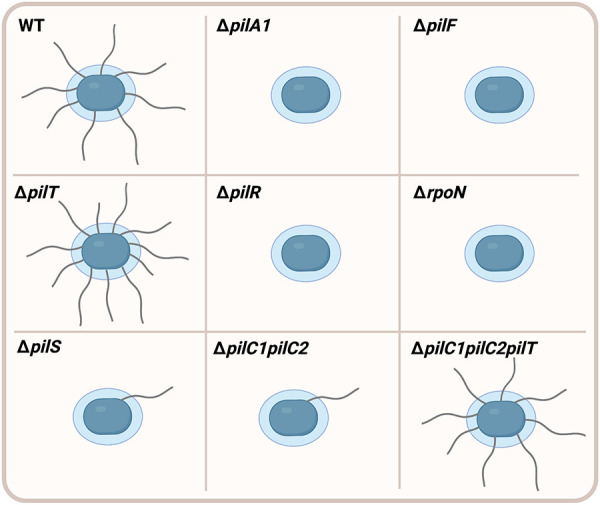
Piliation phenotypes in *K. kingae* mutants. Wild type **(**WT) *K. kingae* bacteria (blue) produce high levels of filamentous surface fibers called type IV pili. Deletion of the *pilA1* gene (Δ*pilA1*) encoding the PilA1 major pilin subunit results in abrogation of surface pili. The pilus fiber is assembled by an assembly ATPase PilF, and deletion of the *pilF* gene (Δ*pilF*) prevents surface piliation. T4P retraction is controlled by the PilT retraction ATPase, and deletion of the *pilT* gene (Δ*pilT)* results in higher levels of surface piliation compared to WT. Expression of the *pilA1* gene is controlled by the PilR/S two-component system and the *σ* factor *σ*54. Deletion of *pilR* (Δ*pilR*) and the gene encoding *σ*54 (Δ*rpoN*) abrogates surface pili. However, deletion of *pilS* (Δ*pilS*) results in very low levels of surface piliation compared to WT. Elimination of both the PilC1 and PilC2 pilus-associated adhesins (Δ*pilC1pilC2*) significantly decreases levels of surface pili compared to WT. However, surface piliation is restored to WT levels following additional deletion of the *pilT* gene (Δ*pilC1pilC2pilT*), suggesting that PilC1 and PilC2 promote surface piliation through counteracting retraction of the filament.

T4P biogenesis is controlled by the assembly ATPase PilF, which is a cytoplasmic protein that assembles PilA1 subunits into the pilus fiber (6). Deletion of the *pilF* gene abrogates surface pili but does not affect production of PilA1 (6, 7). T4P-mediated activities such as twitching motility and natural competence rely on the ability of T4P filaments to retract towards the cell surface. T4P retraction is controlled by the retraction ATPases PilT and PilU, which are cytoplasmic proteins that remove PilA1 subunits from the base of the T4P fiber (11). Disruption of the *pilTU* operon in strain KK03 results in increased levels of surface pili and decreased levels of adherence to epithelial cells, suggesting that pilus retraction is necessary for full-level adherence to human epithelial cells (11). Consistent with the hypothesis that pilus retraction is required for natural competence and twitching motility, disruption of the *pilTU* operon also results in abrogation of both natural competence and twitching motility (7, 11). The piliation phenotypes of specific *K. kingae* mutants are diagrammed in Figure 1.

Two large pilus-associated proteins called PilC1 and PilC2 are essential for pilus biogenesis, twitching motility, natural competence, and adherence to human cells and extracellular matrix (ECM) proteins (6, 7, 12). These proteins belong to a conserved family of T4P-associated proteins, which includes homologs in *Pseudomonas aeruginosa* (PilY1), *Myxococcus xanthus* (PilY1), and the pathogenic *Neisseria* species (PilC1 and PilC2). In contrast to the near identity between the PilC1 and PilC2 proteins in *Neisseria*, the PilC1 and PilC2 proteins in *K. kingae* share limited sequence similarity, with only 7% identity and 16% similarity overall (6). Strains that produce only PilC1 or only PilC2 show no change in surface piliation or adherence to epithelial cells compared to the wild-type strain that produces both proteins (12). However, a strain lacking both PilC1 and PilC2 (Δ*pilC1pilC2*) has a severe defect in surface piliation (Figure 1), suggesting that PilC1 and PilC2 have important functions in pilus biogenesis (6, 7, 12). Elimination of the PilT retraction ATPase in the Δ*pilC1pilC2* strain (Δ*pilC1pilC2pilT*) results in restoration of surface piliation, suggesting that PilC1 and PilC2 promote high levels of surface piliation through counteracting retraction of the pilus filament (7). Although the Δ*pilC1pilC2pilT* strain is piliated, it does not adhere to human epithelial cells, raising the hypothesis that PilC1 and PilC2 have adhesive activity (7). In support of this hypothesis, purified PilC1 and PilC2 bind to human epithelial monolayers in a dose-dependent manner and exhibit saturable binding, demonstrating that these proteins are T4P-associated adhesins (7).

Experiments with mutants producing PilC1 and PilC2 truncations and with purified PilC1 and PilC2 fragments established that the binding activity of PilC1 and PilC2 is localized to the N-terminal region of these proteins (7). Deletion of the N-terminal domain of PilC1 abrogates twitching motility and natural competence (7). In contrast, deletion of the N-terminal domain of PilC2 eliminates twitching motility but has no effect on natural competence (7). Production of the C-terminal region of either PilC1 or PilC2 is sufficient to promote surface piliation but not adherence, suggesting that the C termini of PilC1 and PilC2 are sufficient to promote pilus biogenesis (7). Similar to other members of the PilC family, the C termini region of the *K. kingae* PilC1 and PilC2 proteins contains a predicted *β*-propeller fold and a calcium-binding (Ca-binding) site (12). In contrast to studies of the Ca-binding site in *P. aeruginosa* PilY1, mutation of the Ca-binding sites in *K. kingae* PilC1 and PilC2 has no effect on surface piliation (12). Interestingly, the 9-amino acid Ca-binding site in PilC1 is required for twitching motility and adherence to human epithelial cells, while the 12-amino acid Ca-binding site in PilC2 only has a minor impact on twitching motility and no effect on adherence (12). This work has identified PilC1 and PilC2 as multi-functional T4P-associated adhesins. Further studies are needed to characterize the specific molecular mechanisms that PilC1 and PilC2 utilize to promote T4P-mediated virulence phenotypes.

### *Kingella* NhhA homolog (Knh)

Analysis of a diverse collection of *K. kingae* clinical isolates revealed that a significant number of pharyngeal isolates are nonpiliated (8). As T4P are important for adherence to epithelial cells, Porsch et al. sought to uncover additional factors that may influence *K. kingae* adherence to epithelial cells (11). Examination of a *K. kingae* draft genome sequence for homologs of adhesins in other Neisseriaceae family members uncovered an open reading frame encoding a protein with C-terminal sequence similarity to the *Neisseria meningitidis* NhhA trimeric autotransporter. This *K. kingae* protein was named Knh for Kingella NhhA homolog and was confirmed to be a trimeric autotransporter through demonstration of the trimeric *β*-barrel in the outer membrane when produced in *Escherichia coli* (11). Adherence assays with human epithelial cells demonstrated that Knh is required for full-level adherence, as the adherence level mediated by T4P in the absence of Knh is reduced by approximately half (11). Dynamic adherence assays with shear stress revealed that Knh-mediated adherence is stronger than T4P-mediated adherence (13).

Further work established that the adhesive potential of Knh is dependent on post-translational modification *via* glycosylation (14). *K. kingae* encodes a homolog of the *Haemophilus influenzae* HMW1C glycosyltransferase that N-glycosylates Knh with glucose residues. Without glycosylation, the amount of Knh on the bacterial surface is reduced and adherence to human epithelial cells is abrogated (14).

Through analysis of the amino acid sequence of Knh, it was found that the passenger domain contains two putative adhesive domains (11). The N-terminal region is predicted to encode YadA-like head domains, based on the YadA trimeric autotransporter adhesin of *Yersinia enterocolitica* (15, 16). The head domains of YadA are responsible for the adhesive activity of this trimeric autotransporter, raising the possibility that these predicted domains of Knh may contain adhesive activity as well (11, 15, 16). Additionally, Knh contains an ISneck2 domain with similar architecture to the ISneck1 domain found in the adhesive regions of the *H. influenzae* Hia and Hsf proteins (11, 17). Further studies are necessary to experimentally confirm the adhesive domains of Knh.

### RTX family pore-forming toxin

In order to invade the bloodstream, *K. kingae* must breach the epithelial barrier at the site of colonization. Translocation across the epithelial barrier and entry into the bloodstream likely involves multiple factors, including a repeats-in-toxin (RTX) family protein called RtxA (18). RTX family members have been identified in diverse bacterial pathogens, including *Bordetella pertussis, Escherichia coli, Vibrio cholerae, Vibrio vulnificus, Moraxella spp., Pasteurella spp., Actinobacillus spp.,* and *Aggregatibacter spp.,* among others (19–24). Members of this protein family are defined by the presence of nonapeptide repeats with the consensus sequence X-(L/I/F)-X-G-G-X-G-(N/D)-D in the RTX domain of the protein (25). This repeat sequence binds extracellular calcium, facilitating both folding of RTX family proteins and secretion from the organism (24, 25).

The RtxA toxin was first identified and characterized by Kehl-Fie et al. (18) and is encoded by the *rtxA* gene, which is conserved across all known strains of *K. kingae*. The *rtxA* gene is absent in other *Kingella* species except *K. negevensis* (26–30). RtxA is cytotoxic against a broad range of eukaryotic cells *in vitro*, including red blood cells, epithelial cells (HeLa, A549, FaDu, HLac-78), endothelial cells, synovial cells (SW-982, Hig-82), osteoblasts (U-2), and monocytes (THP-1, RAW264.7) (18, 24, 31–33). Mutants that lack RtxA are non-hemolytic and non-cytotoxic *in vitro* and are avirulent in an infant rat model of invasive disease, underscoring the role of this toxin as a major contributor to *K. kingae* virulence (10, 33).

Cell death is mediated by insertion of RtxA into the host cell membrane, resulting in pore formation (23, 24, 34–36). Unlike related toxins, such as the *B. pertussis* and *E. coli* RTX toxins, RtxA does not target *β*2-integrins in the host cell membrane. Rather, this toxin associates with membrane cholesterol, likely facilitated by a set of cholesterol recognition/interaction amino acid (CRAC) motifs in the N-terminus of the protein (24). Once in the membrane, RtxA oligomerizes, resulting in formation of a cation-selective pore that measures 1.9 nm in diameter, as demonstrated using purified RtxA and planar lipid bilayers (34).

There are five toxin-associated genes that are required for production, activation, and secretion of the *K. kingae* RtxA toxin, designated *rtxB, rtxD, rtxC, rtxA,* and *tolC*. These genes are present in two loci in different regions of the chromosome. In most strains with publicly available genomes, one locus contains *rtxB, rtxD,* and *rtxC* and a second locus contains *rtxC, rtxA,* and *tolC*, resulting in two copies of *rtxC* (23, 28). In *K. kingae* strain KWG-1, one locus contains all five genes, and a second locus contains *rtxC, rtxA,* and *tolC*, resulting in two copies of both *rtxC* and *rtxA.* The prevalence of the KWG-1 configuration in the *K. kingae* population and the phenotypic consequences of the duplication of *rtxA* are unknown. Both *rtx* loci are flanked by genes predicted to be associated with DNA transposition and have a decreased G + C content relative to the rest of the *K. kingae* genome (18). As a consequence, some investigators have hypothesized that the toxin may have been acquired as a part of a horizontal gene transfer event (18, 23, 28).

Little is known about the regulatory mechanisms that govern transcription of *rtxA,* but *rtxA* expression is influenced at least in part by phase variation (37). Phase variation is mediated by the phase variable type III DNA methyltransferase, ModK. Expansion or contraction of a tandem tetranucleotide repeat sequence in *modK* results in a frame shift that introduces a premature stop codon, generating a truncated and inactive ModK protein. Inactivation of ModK results in a subpopulation of organisms with less surface-associated RtxA (37). Phase variation of ModK also influences levels of the surface-associated heat shock proteins DnaK and GroEL.

**Figure 2:**
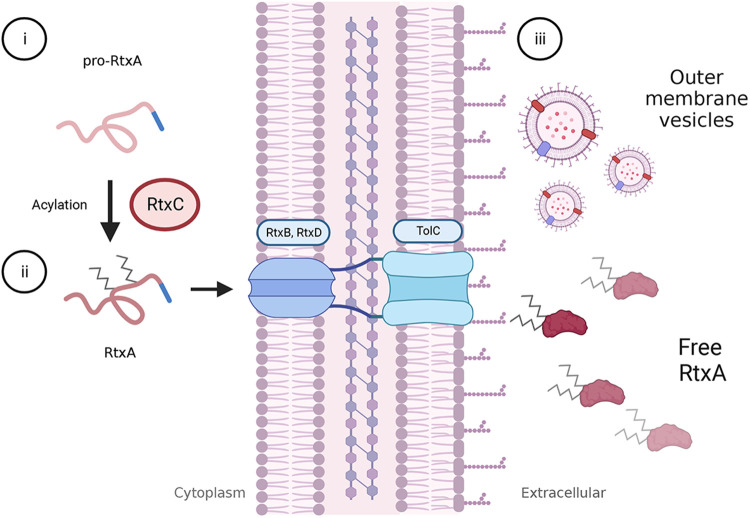
Activation and secretion of RtxA. The *rtxA* gene encodes a pro-toxin in *K. kingae.* Following translation, pro-RtxA remains unfolded and associates with RtxC in the cytosol, possibly near the cell membrane. (i) RtxC catalyzes the addition of an acyl-moiety onto each of two, conserved lysine residues. (ii) As with other RTX toxins, RtxA is thought to remain unfolded until it is exported from the bacterial cell *via* a type I secretion system comprised of RtxD and RtxB in the inner membrane and TolC in the outer membrane. Export is facilitated by a C-terminal secretion signal, and folding is likely mediated through the interaction of extracellular calcium with the RtxA calcium-binding domain. (iii) Outside of the cell, RtxA is found in a free state in spent media as well as in association with outer membrane vesicles (OMVs). It is unclear if the OMV-associated toxin is within the OMV or associated with the membrane. Both free RtxA and OMV-associated RtxA can cause host cell lysis.

RtxA is synthesized as a pro-toxin, which is non-cytotoxic and non-hemolytic. The pro-toxin is activated by the RtxC protein, which adds two acyl-chain moieties that play a key role in facilitating RtxA insertion into the host cell membrane. The activated RtxA toxin is then secreted by a type I secretion system (TISS) that is comprised of RtxB, RtxD, and the TolC outer membrane protein. The proposed secretion pathway for RtxA is shown in Figure 2 (18, 24, 25, 36).

Beyond secretion *via* the TISS, RtxA is released from the organism in outer membrane vesicles (OMV). OMV production is highly variable among clinical isolates (31, 32). Along with RtxA, *K. kingae* OMVs contain a host of other proteins in the outer membrane. OMVs are rapidly phagocytosed by eukaryotic cells and are cytotoxic against red blood cells and monocytes but not epithelial cells (32). Exposure to OMVs results in increased production of granulocyte-macrophage colony-stimulating factor (GM-CSF), interleukin-6 (IL-6), IL-1β, and tumor necrosis factor-α (TNF-α) from SW982 (synovial) cells and hFOB 1.19 (osteoblast) cells *in vitro* (31, 32). Currently, it is unclear if stimulation of these inflammatory molecules is due to RtxA or to other factors found in OMVs, such as lipopolysaccharide (LPS).

### Polysaccharide capsule

Polysaccharide capsules are produced by many pathogenic bacteria and promote bacterial survival in diverse environments, providing protection against a variety of extracellular insults. Capsules are surface-exposed, lipid-anchored, highly-hydrated polymers that promote bacterial survival within a host by assisting in the evasion of host immune mechanisms, including phagocytosis and complement-mediated killing, making them attractive targets as vaccine antigens (38, 39).

The hypothesis that *K. kingae* is encapsulated began with qualitative examination of the mucoid colony phenotype on chocolate agar, a phenotype that is common in other bacteria that produce a polysaccharide capsule (11). A search through the draft genome sequence of *K. kingae* for homologs of genes responsible for encapsulation in other organisms led to discovery of a locus with similarity to the ABC-type capsule export operon in *N. meningitidis*, containing the *ctrABCD* genes. Thin-section transmission electron microscopy after staining with ferritin and visualization of Alcian blue-stained acid extracts confirmed that *K. kingae* produces a surface-associated polysaccharide capsule that is absent when *ctrA* is disrupted (11).

Further homology-based approaches and a transposon mutagenesis screen identified additional capsule synthesis and assembly genes in *K. kingae*. These include genes homologous to the *N. meningitidis lipB* and *lipA* genes and the *E. coli kpsS* and *kpsC* genes involved in synthesizing the poly *β*-Kdo linker between the capsular polysaccharide and the lipid anchor (11, 40–42) Individual disruption of the *K. kingae lipB* or *lipA* homolog results in loss of encapsulation and accumulation of capsular polysaccharide in the cytoplasm, as evidenced by the presence of lacunae in thin section electron micrographs (42). These results are consistent with studies in *E. coli* by Willis and Whitfield, demonstrating that KpsS adds a single *β*-Kdo residue onto a lipid moiety (phosphatidylglycerol) on the cytoplasmic side of the inner membrane and KpsC then incorporates additional *β*-Kdo residues onto the first residue (41). The transposon mutagenesis screen also identified a predicted glycosyltransferase gene necessary for capsule production in strain KK01, a stable nonspreading/noncorroding derivative of septic arthritis isolate 269–492, and designated *csaA*. Targeted deletion of *csaA* results in a complete absence of both intracellular and extracellular capsular polysaccharide, suggesting that this gene is involved in capsule synthesis (42). In contrast to other encapsulated organisms, the *K. kingae ctrABCD*, *lipA*, *lipB*, and *csaA* genes are not present at a single locus in the chromosome and instead are scattered throughout the genome, raising questions about the regulatory mechanism used to control capsule biosynthesis and export (42, 43).

**Figure 3:**
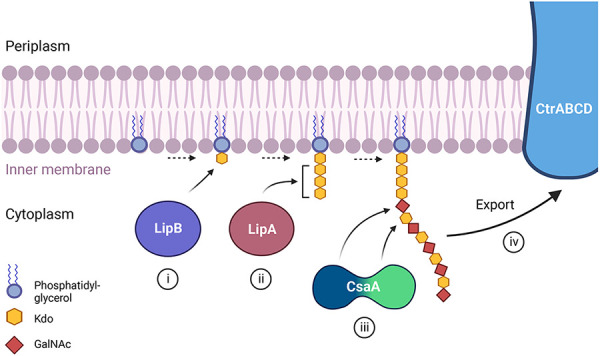
A proposed model depicting the role of each of the known capsule biosynthesis gene products in *K. kingae* strain KK01 producing capsule type a. (i) The *lipB* gene encodes a *β*-Kdo transferase that adds a single *β*-Kdo residue onto a lipid moiety (phosphatidylglycerol) at the cytoplasmic side of the bacterial membrane. (ii) The *lipA* gene encodes a *β*-Kdo transferase that adds multiple *β*-Kdo residues onto the initial *β*-Kdo residue and creates a short *β*-Kdo chain. (iii) The *csaA* gene encodes a bifunctional enzyme with a GalNAc transferase at the N-terminal region (blue portion) and a Kdo transferase in the middle and C-terminal region (green portion) of the protein. CsaA builds the capsular polysaccharide chain composed of GalNAc and Kdo repeating units onto the chain of *β*-Kdo residues, with the N-terminal region (blue) adding GalNAc and the middle and C-terminal region (green) adding Kdo. (iv) The capsular polysaccharide is then shuttled to the outer membrane through the ABC-type capsule transporter encoded by *ctrABCD*.

To determine the composition and structure of the *K. kingae* polysaccharide capsule, glycosyl composition analysis and NMR spectroscopy were performed on purified capsular polysaccharide from strain KK01 (44). These studies established that the polysaccharide is composed of N-acetyl galactosamine (GalNAc) and 3-deoxy-D-manno-oct-2-ulosonic acid (Kdo), with the structure [3)-*β*-Gal*p*NAc-(1→5)-*β*-Kdo*p*-(2→] (44). Point mutations in the *csaA* capsule synthesis gene revealed that CsaA is a bifunctional enzyme (42). The N-terminal region contains a putative GalNAc transferase domain, and the middle and C-terminal region contains a putative *β*-Kdo transferase domain. This glycosyltransferase creates a polymer of GalNAc and Kdo and is able to create both the *β*-Gal*p*NAc-(1→5)-*β*-Kdo*p* linkage and the *β*-Kdo*p*-(2→3)-*β*-Gal*p*NAc linkage, linking the polysaccharide to the terminal *β*-Kdo residue of the poly *β*-Kdo linker and synthesizing the capsular polysaccharide (42). The interplay of each of the capsule biosynthesis gene products in strain KK01 is summarized in Figure 3.

The structure of the strain KK01 capsular polysaccharide is similar to a previously reported surface polysaccharide with the structure [6)-*α*-D-Glc*p*NAc-(1→5)-*β*-Kdo*p*-(2→] in *K. kingae* strain PYKK181 (45). With the hypothesis that the strain PYKK181 surface polysaccharide represents a distinct capsule type, Starr et al. used a PCR approach to screen a collection of 417 Israeli invasive and carrier isolates for the presence of additional capsule synthesis loci (43). Four distinct capsule synthesis loci were discovered, producing four different capsular polysaccharide types. The *csa* locus produces the GalNAc-Kdo capsule identified in strain KK01, designated capsule type a (42, 43). The *csb* locus produces the GlcNAc-Kdo capsule present in strain PYKK181, designated capsule type b (43, 45). The *csc* locus produces a capsule composed of ribose and Kdo with the structure [3)-*β*-D-Rib*f*-(1→2)-*β*-D-Rib*f*-(1→2)-*β*-D-Rib*f*-(1→4)-*β*-Kdo*p*-(2→], designated capsule type c (43). The *csd* locus produces a capsule composed of galactose and GlcNAc with the structure [P-(O→3)[*β*-D-Gal*p*-(1→4)]-*β*-D-Glc*p*NAc-(1→3)-*α*-D-Glc*p*NAc-1-], designated capsule type d (43). The type a capsule is identical to the capsule of *Moraxella nonliquefacians* strain 3828/60, a commensal in the human upper respiratory tract (46), and the type b capsule is identical to the serotype 5a capsule of *Actinobacillus pleuropneumoniae*, a respiratory porcine pathogen (43, 45, 47). The capsule type c and d structures are novel (43).

The analysis of Israeli invasive and carrier isolates revealed that the type a and type b capsules are enriched in invasive isolates, accounting for >95% of these isolates. In contrast, the type c and type d capsules are disproportionally present in carrier isolates (43). Analysis of an international collection of isolates from the United States, Canada, Iceland, France, Switzerland, Spain, and New Zealand also revealed dominance of the type a and type b capsule types among invasive isolates (48–50). Whether the type a and type b capsules directly confer greater pathogenicity or are instead associated with other virulence determinants is an active area of investigation.

Detailed studies have implicated the *K. kingae* capsule as a key determinant of *K. kingae* pathogenicity. As described in the sections above, colonization of the oropharynx is presumed to be initiated by adherence of the bacterium to oropharyngeal epithelial cells. Studies have shown that the capsule masks the Knh adhesin and interferes with Knh-mediated high-affinity adherence to human epithelial cells when T4P retraction is lacking (11, 13). Examination of *K. kingae* interactions with human epithelial cells by transmission electron microscopy using ferritin staining to highlight the capsule suggests that T4P retraction pulls the bacterium into close contact with the host cell, resulting in physical displacement of the bacterial capsule through the retractile force of the fibers and the subsequent exposure of Knh to the surface of host cells (11, 13). This capsule displacement is necessary for Knh-mediated adherence, as the capsule is deeper than Knh is long (13).

In order to survive in the bloodstream and disseminate to sites of invasive infection, *K. kingae* must evade potent immune mechanisms in the intravascular space. The *K. kingae* capsule resists human serum-mediated killing by blocking deposition of IgG and IgM antibodies and the C3b and C4b complement fragments on the bacterial surface and inhibiting activation of the classical complement pathway (51). To delineate whether capsule type influences the ability of *K. kingae* to resist serum-mediated killing, isogenic mutants that produce each of the four capsule types were tested in serum-bactericidal assays. These studies revealed that the presence of any of the four capsule types is sufficient to promote serum survival under *in vitro* conditions (51). Consistent with the *in vitro* results, the capsule is necessary for full virulence in the juvenile rat model of invasive disease (42, 51). Depletion of complement components C3 and C5 in the juvenile rats with cobra venom factor prior to infection restores full virulence of unencapsulated mutants, demonstrating the importance of resisting complement for virulence in the rat infection model (51).

Beyond mediating resistance to complement-mediated killing, the polysaccharide capsule plays a key role in resisting neutrophil killing. Using freshly purified human neutrophils, Muñoz et al. demonstrated that the capsule interferes with bacterial binding to neutrophils and prevents neutrophil activation and production and release of neutrophil reactive oxygen species (52).

### Galactan exopolysaccharide

**Figure 4:**
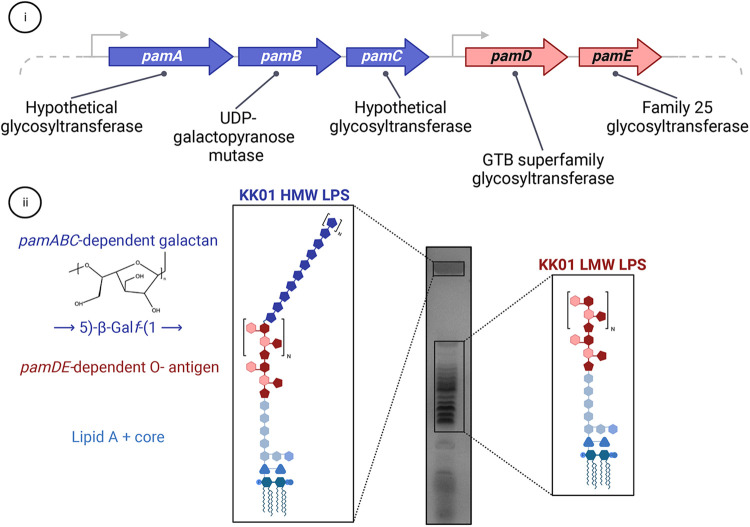
The relationship between the galactan exopolysaccharide and LPS in *K. kingae*. (i) The *pamABCDE* locus is essential for presentation of the galactan exopolysaccharide on the bacterial surface and contains genes encoding four predicted glycosyltransferases (*pamA, pamC, pamD,* and *pamE*) and one predicted UDP-galactopyranose mutase (*pamB*). (ii) The *pamABC* genes are necessary for synthesis of the galactan homopolymer with the structure of →5)-β-Gal*f*-(1→, which is linked to the atypical O-antigen. The *pamDE* genes are required for synthesis of the atypical O-antigen. In wild type *K. kingae* strain KK01, the LPS migrates as two distinct modal clusters: a high molecular weight (HMW) band that contains LPS decorated with both the atypical O-antigen and galactan and a low molecular weight ladder cluster that contains LPS decorated with only the atypical O-antigen.

In addition to the polysaccharide capsule, *K. kingae* was found to have a distinct exopolysaccharide called galactan in cell-free extracts. Work by Bendaoud et al. demonstrated that these cell-free extracts had surfactant-like activity and inhibited biofilm formation by *K. kingae* and several phylogenetically diverse bacterial and fungal pathogens when coated onto an abiotic surface (45). Preliminary characterization of the extracts suggested that they contained a high abundance of DNA, prompting the name poly-DNA-containing anti-adhesive material extract or PAM extract. Selective removal of PAM extract components from *K. kingae* strain PYKK181 suggested that the bioactive component was carbohydrate-based, and NMR analyses revealed a galactofuranose homopolymer, or galactan, with the structure →3)-β-D-Gal*f*-(1→6)-β-D-Gal*f*-(1→ (45). This study also identified a five gene locus designated *pamABCDE,* encoding a predicted UDP-galactopyranose mutase (enzyme required to catalyze the conversion of UDP-galactopyranose to UDP-galactofuranose) and four putative glycosyltransferases (Figure 4A). Expression of the *pam* genes in *E. coli* established that production of the galactan exopolysaccharide was dependent on only the *pamABC* genes (45).

In addition to the →3)-β-D-Gal*f*-(1→6)-β-D-Gal*f*-(1→ galactan isolated from strain PYKK181, a →5)-β-Gal*f*-(1→ galactan has been isolated from *K. kingae* strain KK01. These are currently the only two documented galactan structures in *K. kingae,* and more studies are needed to appreciate the diversity of these galactan structures across the *K. kingae* population structure.

Functional studies performed with strain KK01 have established that the galactan exopolysaccharide has a critical role in virulence. In particular, the galactan mediates resistance to complement-mediated serum killing, independent of the polysaccharide capsule (51). The galactan also mediates resistance to neutrophil phagocytosis and killing, again independent of the polysaccharide capsule (52). In the infant rat model of systemic *K. kingae* disease, mutants lacking the galactan are significantly attenuated in virulence, and mutants lacking the capsule and galactan are avirulent (51). These functions suggest that galactan must be tethered to the bacterial surface by some mechanism. Recent work has revealed that surface tethering of the galactan is dependent on the *K. kingae* lipopolysaccharide (LPS) atypical O-antigen (53).

LPS is a highly abundant glycolipid that is associated with the gram-negative bacterial outer membrane and promotes multiple functions related to barrier integrity and interactions with the environment (54). LPS is a tripartite molecule composed of lipid A, which is anchored in the outer membrane, a core oligosaccharide composed of non-repeating sugar residues, and in some organisms an O-antigen composed of repeating sugar units that vary greatly across bacterial species (54, 55). Initial characterization of the *K. kingae* LPS revealed that it varies greatly from the LPS of related species. Notably, resolution of purified LPS from wild type *K. kingae* revealed two modal clusters, including a low molecular weight (LMW) cluster that displays a ladder migration pattern and a high molecular weight (HMW) cluster that contains an additional polysaccharide component (Figure 4B) (53). The uniformity of the ladder migration pattern observed in the LMW LPS species suggested the presence of a repeating sugar unit, implying that *K. kingae* produces an atypical O-antigen. Glycosyl composition analysis and 1-D NMR revealed that the additional polysaccharide component of the HMW LPS is the galactan exopolysaccharide, implying a novel structural association between the *K. kingae* LPS and galactan. Homology-based approaches indicated that the *pamD* and *pamE* gene products contain folds that are shared with the GTB superfamily of glycosyltransferases and the Family 25 glycosyltransferases, respectively. Some proteins in other organisms that contain these folds are involved in LPS biosynthesis. The homology analysis of the *pamD* and *pamE* gene products and the location of the *pamD* and *pamE* genes immediately adjacent to the *pamABC* genes suggested that these gene products might function in *K. kingae* LPS biosynthesis and influence the structural association between the LPS and galactan (Figure 4A) (53). Deletion of *pamD* and *pamE* resulted in production of a truncated LPS lacking both the LMW and HMW LPS species, highlighting the essential role of the *pamD* and *pamE* genes in biosynthesis of the *K. kingae* atypical O-antigen (53). Furthermore, the loss of the HMW LPS species in *K. kingae* strains lacking the *pamD* and *pamE* genes established that surface anchoring of the galactan requires the atypical O-antigen (53).

Recent studies have begun to elucidate how the structural linkage between the LPS O-antigen and the galactan exopolysaccharide influences the function of both surface polysaccharides. In particular, initial characterization of the galactan exopolysaccharide suggested that it was able to promote resistance to cationic antimicrobial peptides (CAMPs), including polymyxin B and HNP-1 (52). However, more recent work examining mutants lacking the *pamABC* genes and deficient in galactan/HMW LPS production retained resistance to polymyxins, indicating that the galactan on its own is dispensable for CAMP resistance (53). Conversely, mutants lacking the *pamDE* genes were significantly more susceptible to polymyxins, suggesting that the LMW LPS O-antigen is a primary driver of CAMP resistance in *K. kingae* (53).

Identification of the novel structural relationship between the *K. kingae* LPS and galactan molecules has provided a mechanism for surface anchoring of the galactan exopolysaccharide and for the unique functionality of this exopolysaccharide in interactions with host immune components. More studies are needed to understand the individual roles of the *pamABCDE* genes in galactan and LPS biosynthesis, specifically ligation of the galactan to the growing LPS molecule and the spatiotemporal dynamics of LPS and galactan assembly and surface translocation.

### Genomics

At the time of this publication, genomes for 57 strains of *K. kingae* are publicly available. Genomes range in size from 1.95 megabases (ATCC23330) to 2.14 megabases (KWG-1), with an average of ∼2,000 predicted open reading frames per genome. *K. kingae* is closely related to *K. negevensis,* with a number of shared virulence factors, including type IV pili, Knh, RtxA, a polysaccharide capsule, and a galactan exopolysaccharide (28–30). Amit et al. performed pulsed field gel electrophoresis (PFGE) on a collection of 181 invasive *K. kingae* isolates collected between 1991 and 2012 in Israel and identified 32 different clones, with five clones (clones B, H, K, N, and P) accounting for more than 70% of the invasive isolates (56). Clones K, N, and P were significantly associated with bacteremia, skeletal infections, and endocarditis, respectively, potentially reflecting different tissue tropisms (56). This study further categorized the population by applying a sequence typing scheme using the genes *abcZ, adk, aroE, cpn60, gdh,* and *recA* (56). Subsequent studies have identified 73 global sequence types (STs) comprising 12 distinct complexes (STcs). Five of these STcs account for 72% of global isolates, though their distribution is skewed based on geographic location (56–58).

As stated above, all known isolates of *K. kingae* contain the *rtxA* gene, which has been used as a molecular target for the clinical diagnosis of invasive *K. kingae* disease (28, 58, 59). In addition, all isolates contain the genetic machinery for polysaccharide capsule export, galactan exopolysaccharide biosynthesis, and type IV pili biogenesis. Production of functional T4P is critical for *K. kingae* natural transformation, which is presumed to be a major contributor to *K. kingae* genomic diversity (7). Natural transformation by exogenous DNA is influenced in *K. kingae* by the presence of a DNA uptake sequence (DUS), which is shared with *Kingella denitrificans*, but exists in different dialects in many other Neisseriaceae family members (60). A novel, rapid, PCR-based typing strategy based on the *K. kingae* DUS has been shown to correctly classify *K. kingae* isolates into MLST-derived STcs (57).

In addition to DNA acquisition *via* natural transformation, at least one conjugative plasmid was originally identified in strain KWG-1 (61). This plasmid has been named ISkk1, is thought to be integrated into the genome, and encodes a type IV secretion system. Additionally, ISkk1 contains several antibiotic resistance cassettes, including cassettes that encode resistance to *β*-lactams, sulfonamides, streptomycin, and tetracycline (61). The presence of a *β*-lactamase has been noted in other isolates from the United States, Israel, and Iceland (62). Based on a study of Israeli invasive and carrier isolates, ∼10% of carrier isolates (belonging to the A, F, and *Ψ* PFGE groups) and ∼1% of invasive isolates, produce a *β*-lactamase (62). Beyond ISkk1, little is known about the pangenome of *K. kingae* (62–65).

## Conclusions

The advances in our understanding of *K. kingae* virulence factors detailed in this review have revealed multiple bacterial surface factors implicated in the pathogenicity of this fastidious bacterium. Several key findings have identified targets for therapeutic intervention. For example, the fact that greater than 95% of invasive disease isolates express either the type a or type b capsule raises the possibility that a glycoconjugate vaccine incorporating these two polysaccharides may be an effective approach to prevent invasive disease given the success of capsule-based conjugate vaccines in preventing morbidity and mortality due to other bacteria such as *H. influenzae* type b, *N. meningitidis*, and *Streptococcus pneumoniae*. In addition, it is intriguing to speculate that targeting type IV pili or Knh may be an effective strategy to prevent *K. kingae* colonization of the oropharynx. As we continue to gain a more complete picture of the pathogenesis of *K. kingae* disease, additional strategies for prevention and treatment of disease are likely to emerge.
